# Lubiprostone Decreases the Small Bowel Transit Time by Capsule Endoscopy: An Exploratory, Randomised, Double-Blind, Placebo-Controlled 3-Way Crossover Study

**DOI:** 10.1155/2014/879595

**Published:** 2014-12-29

**Authors:** Mizue Matsuura, Masahiko Inamori, Hiroki Endo, Tetsuya Matsuura, Kenji Kanoshima, Yumi Inoh, Yuji Fujita, Shotaro Umezawa, Akiko Fuyuki, Shiori Uchiyama, Takuma Higurashi, Hidenori Ohkubo, Eiji Sakai, Hiroshi Iida, Takashi Nonaka, Seiji Futagami, Akihiko Kusakabe, Shin Maeda, Atsushi Nakajima

**Affiliations:** ^1^Hepatology and Gastroenterology, Yokohama City University Hospital, Kanagawa, Yokohama 236-0004, Japan; ^2^Office of Postgraduate Medical Education, Yokohama City University Hospital, 3-9 Fukuura, Kanazawa-ku, Kanagawa, Yokohama 236-0004, Japan; ^3^Division of Gastroenterology, Nippon Medical School, Tokyo 113-8603, Japan; ^4^Department of General Medicine, Yokohama City University School of Medicine, Kanagawa, Yokohama 236-0004, Japan; ^5^Department of Gastroenterology, Yokohama City University Hospital, Kanagawa, Yokohama 236-0004, Japan

## Abstract

The aim of this study was to investigate the usefulness of lubiprostone for bowel preparation and as a propulsive agent in small bowel endoscopy. Six healthy male volunteers participated in this randomized, 3-way crossover study. The subjects received a 24 *μ*g tablet of lubiprostone 60 minutes prior to the capsule ingestion for capsule endoscopy (CE) and a placebo tablet 30 minutes before the capsule ingestion (L-P regimen), a placebo tablet 60 minutes prior to CE and a 24 *μ*g tablet of lubiprostone 30 minutes prior to CE (P-L regimen), or a placebo tablet 60 minutes prior to r CE and a placebo tablet again 30 minutes prior to CE (P-P regimen). The quality of the capsule endoscopic images and the amount of water in the small bowel were assessed on 5-point scale. The median SBTT was 178.5 (117–407) minutes in the P-P regimen, 122.5 (27–282) minutes in the L-P regimen, and 110.5 (11–331) minutes in the P-L regimen (*P* = 0.042). This study showed that the use of lubiprostone significantly decreased the SBTT. We also confirmed that lubiprostone was effective for inducing water secretion into the small bowel during CE.

## 1. Introduction

Capsule endoscopy (CE) has been established as a convenient method for the evaluation of the small bowel. CE provides a higher diagnostic yield than barium contrast radiography of the small bowel or enteroscopy [1–4]. It is safe, painless, and well-tolerated [5]. Despite these advantages, the diagnostic yield of CE may be restricted by some limitations, including technical difficulties, inability of some patients to swallow the capsule, the relatively poor quality of the small bowel images, and the frequent inability of this modality to allow complete assessment of the small bowel; in almost 10–15% of the cases, the capsule does not reach the cecum within the imaging period [6]. In addition, the overall results differ among studies, with the reported percentage of cases of incomplete visualization of the mucosal surfaces due to bubbles, or luminal residue obscuring the view, especially in the distal small bowel, varying from 5 to 30% [7–10].

Several studies have examined the possibility of shortening the transit time and improving the bowel cleanness by using different medications for bowel preparation and prescribing different fasting periods [11, 12]. The current bowel preparation protocols, although still not standardized, usually include clear liquids on the day before and nothing by mouth within 8 to 10 hours before the capsule ingestion. It has been reported that this method resulted in inadequate small bowel preparation in 30% of the study subjects. Comparisons of this type of preparation with gut lavage or oral sodium phosphate regimens have shown similar efficacies of all the regimens [13]. Therefore, the optimal preparation method for small bowel CE has not yet been established.

Lubiprostone (Amitiza; Takeda Pharmaceuticals North America, Deerfield, IL) selectively activates the type-2 chloride channels in the apical membrane of the GI epithelium, inducing net fluid secretion. It is currently approved by the U.S. Food and Drug Administration for the treatment of chronic idiopathic constipation and constipation-predominant irritable bowel syndrome. The proposed primary mechanism of action of lubiprostone in the gastrointestinal tract is increased chloride ion transport into the intestinal lumen by the drug caused by the opening of ClC-2, which results in increased intestinal secretion and accelerated mass transit [14, 15]. Transit time studies by Camilleri et al. [16] revealed that lubiprostone accelerated the small bowel transit and colonic transit times. A previous clinical study performed in healthy volunteers showed that lubiprostone accelerated intestinal transit, increased fasting gastric volume, and delayed gastric emptying [16].

We designed an exploratory double-blind, placebo-controlled 3-way crossover trial to investigate the usefulness of lubiprostone, both as a bowel preparation agent and as a propulsive agent for small bowel endoscopy.

## 2. Methods

### 2.1. Trial Design

An exploratory, randomised, double-blind, placebo-controlled 3-way crossover study.

### 2.2. Trial Registration

Trial registry is the University Hospital Medical Information Network Clinical Trials Registry (UMIN-CTR), UMIN000010965, registered in 26 July 2013.

### 2.3. Participant

The study was performed between June 2013 and September 2013 at Yokohama City University School of Medicine. The subjects were 6 asymptomatic male volunteers (average age: 35.8 years; age range: 29–50 years), recruited by a study nurse. Baseline evaluations included a medical history, physical examination, and collection of demographic data (Table 1).

The exclusion criteria were history of gastric or intestinal surgery, clinical or suspected motility disorders of the stomach, age <20 years, history of intake of medications during the previous week that could potentially affect the gastrointestinal motility, clinical or suspected drug allergy, and clinical or suspected malignancy.

### 2.4. Intervention

**Figure 1:**
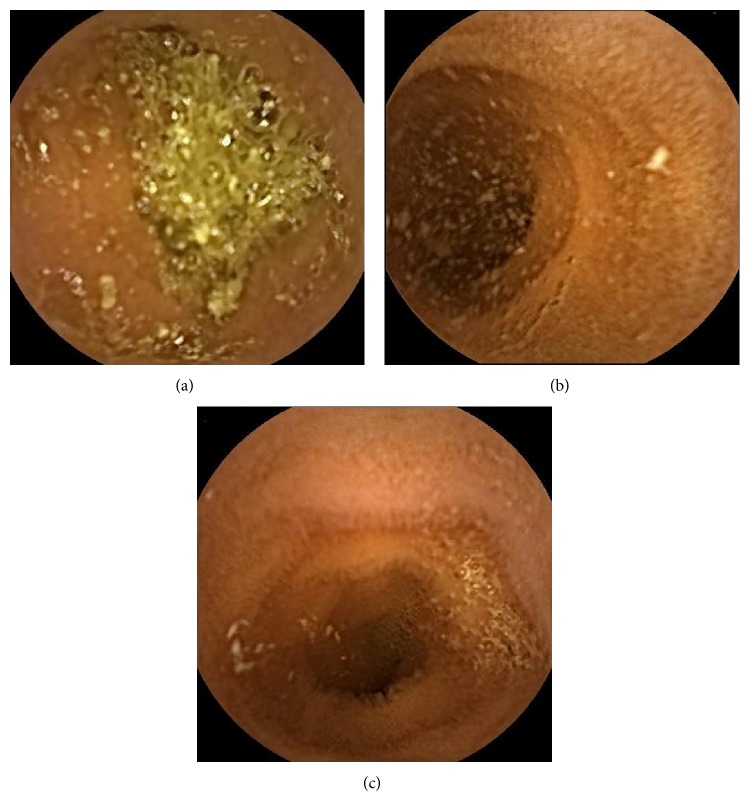
Typical images of each regimen. (a) P-P regimen, (b) L-P regimen, and (c) P-L regimen. (a) showed usual image. (b) and (c) showed increased amount of water in small intestine.

This was a randomized, double-blind, placebo-controlled, 3-way crossover study of subjects who volunteered to undergo CE. In all the subjects, the CE was performed with the PillCam SB2 CE system (Given Imaging Ltd.), and the images were viewed with the Rapid 5 Reader. The subjects were randomly assigned to receive a 24 *μ*g tablet of lubiprostone 60 minutes prior to the capsule ingestion for CE and a placebo tablet 30 minutes before the capsule ingestion (L-P regimen), a placebo tablet 60 minutes prior to the capsule ingestion for CE and a 24 *μ*g tablet of lubiprostone 30 minutes prior to the capsule ingestion (P-L regimen), or a placebo tablet 60 minutes prior to the capsule ingestion for CE and a placebo tablet again 30 minutes prior to the capsule ingestion (P-P regimen) (Figure 1). Each of the test conditions was separated by a washout period of at least 7 days.

### 2.5. CE Procedure

All the study subjects were instructed to have a light breakfast and then clear liquids on the day prior to the CE. Furthermore, they were instructed to have nothing by mouth for at least 8 hours prior to the capsule ingestion for CE. Lubiprostone or placebo was administered 60 minutes and 30 minutes prior to the capsule ingestion in accordance with the protocols described above. The PillCam Small Bowel CE system (Given Imaging, Yokneam, Israel) with the PillCam SB2 capsule and Rapid 5 software platform were used for the study. All the CE images were read by two investigators (Masahiko Inamori and Mizue Matsuura ), both of whom were blinded to the group allocation status of the subjects. The small bowel examination was considered to be complete if the capsule had passed into the colon.

### 2.6. Gastric and Small Bowel Transit Times

The gastric transit time (GTT) was calculated from the time the capsule entered the stomach until it crossed the pylorus. Small bowel transit time (SBTT) was determined as the time from the first duodenal image until the capsule entered the colon and could be calculated only in cases in which the capsule had reached the colon.

### 2.7. Adequacy of Bowel Preparation

The quality assessment of the capsule endoscopic images was made in accordance with the scale used by Postgate et al., with some modification [15]. We used a 5-point scale (0–4) based on the percentage of the capsule images that were unimpaired by the presence of debris or dark luminal fluid (4, 100–80%; 3, 80–60%; 2, 60–40%; 1, 40–20%; 0, 20–0%). The average scores for 5 min segments of the video were assessed from capsule entry into the proximal duodenum (0% of the SBTT) and for every 10% of the SBTT thereafter, with the score for the final segment recorded in the terminal ileum (100% of the SBTT).

### 2.8. Assessment of the Amount of Water in the Small Bowel

We used a 5-point scale (0–4) based on the percentage of the capsule endoscopy images that showed clear water (4, 100–80%; 3, 80–60%; 2, 60–40%; 1, 40–20%; 0, 20–0%). The average scores for 5 min segments of the video were assessed from capsule entry into the proximal duodenum (0% of the SBTT) and for every 10% of the SBTT thereafter, with the score for final segment recorded in the terminal ileum (100% of the SBTT).

### 2.9. Ethical Approval

The study was conducted in accordance with the Declaration of Helsinki. The study protocol was approved by the Ethics Committee of Yokohama City University Hospital. All the subjects provided their written informed consent.

### 2.10. Statistical Analysis

Statistical evaluation was performed using the Friedman test and Wilcoxon's signed-rank test. The level of significance was set at *P* < 0.05. All statistical analyses were performed with EZR (Saitama Medical Center, Jichi Medical University), which is a graphical user interface for R (The R Foundation for Statistical Computing). In other words, it is a modified version of the R commander designed to add statistical functions frequently used in biostatistics [17].

### 2.11. Outcome

The main outcomes were gastric transit time (GTT) and small bowel transit time (SBTT). Secondary outcomes were adequate cleansing and the amount of water in the small bowel.

## 3. Results

The study was completed in six male subjects (mean age: 39.5 years; range: 29–50 years). The subjects' heights and weights were as follows: mean height, 172.5 cm (height range: 165–178 cm); mean weight, 61.8 kg (weight range: 52–70 kg) (Table 1). No adverse events occurred during the study. All subjects enrolled in the study received placebo and/or lubiprostone and swallowed the SB2 capsule, with the endoscopic images recorded for 8 hours. The median GTT was 22.5 (9–160) minutes in the P-P regimen, 40 (4–122) minutes in the L-P regimen, and 57.5 (15–78) minutes in the P-L regimen (*P* = 0.846). The median SBTT was 178.5 (117–407) minutes in the P-P regimen, 122.5 (27–282) minutes in the L-P regimen, and 110.5 (11–331) minutes in the P-L regimen (*P* = 0.042). The median SBTT values for the L-P and P-L regimens were statistically significantly different from the SBTT in the P-P regimen. The data are summarized in Table 2.

The image quality score was 2.88 ± 1.35 in the P-P regimen, 3.56 ± 0.56 in the L-P regimen, and 3.76 ± 0.85 in the P-L regimen (*P* < 0.001). The amount of water in the small bowel was 1.66 ± 1.65 in the P-P regimen, 3.13 ± 1.64 in the L-P regimen, and 2.60 ± 1.29 in the P-L regimen (*P* < 0.01). The data are summarized in Table 3.

There were no cases of capsule retention or serious adverse events in this study.

## 4. Discussion

This study was designed to evaluate the effect of lubiprostone on the capsule transit time through the GI lumen and its effectiveness as a bowel preparation agent for improving the quality of capsule imaging of the small bowel. Lubiprostone improved the imaging quality of the small bowel as compared to placebo and also improved the SBTT.

Lubiprostone is a PGE1 derivative that is approved for the treatment of chronic constipation and constipation-predominant irritable bowel syndrome. The proposed primary mechanism of action is an increase in the chloride ion transport into the intestinal lumen caused by opening the novel ClC-2 channels, leading to increased intestinal secretion and accelerated mass transit. Among the advantageous characteristics of lubiprostone are its specificity for ClC-2 channels and its lack systemic prostaglandin effects, despite its structural similarity to lubiprostones.

The GTT following administration of lubiprostone was similar to that after administration of placebo. Our findings differ from those of the study reported by Camilleri et al. [16], who reported finding evidence of delayed gastric emptying following the administration of lubiprostone. The main side effect of lubiprostone was nausea, possibly related to delayed gastric emptying. In phase II trials, nausea was reported in as many as 33% of patients receiving 48 *μ*g of lubiprostone daily [18]. Nausea was the most common side effect of lubiprostone, reported in up to 31% of patients in one study [19]. Several possible explanations for nausea have been suggested, including delayed gastric emptying, small intestinal distention secondary to increased gastric secretion, change in gastrointestinal sensation, and/or additional actions of lubiprostone on the gastrointestinal motility [16]. However, the precise mechanism of nausea associated with lubiprostone remains unclear.

Lubiprostone improved the SBTT as compared to placebo. Camilleri et al. showed lubiprostone decreased SBTT in 2006 [16]. In addition, lubiprostone has been shown to accelerate overall colonic transit without significantly changing the rate of emptying of the ascending colon [14]. With the proximal colon likely reabsorbing the increased fluid load from the small intestine, it is postulated that a primary motor effect on the colon beyond the ascending portion may be responsible for this effect. Similarly, the presence of a possible direct smooth muscle effect of lubiprostone on the rest of the gastrointestinal tract has also been suggested.

The use of polyethylene glycol (PEG) before capsule administration has yielded mixed results on intestinal propulsion and the bowel preparation efficacy. There are 2 reports of studies in which PEG was given after the capsule administration for CE, and both studies showed promising results. Fireman et al. [20] reported retrospectively that patients who received 1.5 L of PEG 12 hours before capsule ingestion and 1.5 L of PEG 1 hour after capsule ingestion showed significant shortening of the transit time through the stomach and small bowel as compared to the patients who were bowel-prepared with sodium phosphate and those with no colon preparation. Endo et al. [21] used a standard liquid diet and nothing by mouth for initial preparation and gave patients 500 mL of PEG, 30 minutes after the capsule ingestion. Administration of PEG after capsule ingestion resulted in an increased rate of cecal entry of the capsule and improved distal small bowel imaging.

It has been reported that the use of prokinetics such as metoclopramide [22], erythromycin [12], and mosapride [23] may decrease the randomness in the rate of gastric emptying and reduce the SBTT. Selby [22] reported that the administration of oral metoclopramide before capsule administration reduced the GTT with no effect on the SBTT, but still having a positive effect by increasing the percentage of capsules reaching the cecum. Metoclopramide, with the addition of senna and citrate of magnesia for bowel preparation, has also been shown to improve both the GTT and SBTT. Metoclopramide has several actions that may account for its favorable influence on the capsule transit time. Its main effect is in the proximal gastrointestinal tract. It improves the gastric tone and peristalsis, relaxes the pyloric sphincter, and improves antroduodenal coordination [24] by a combination of its cholinergic and antidopaminergic effects [25].

The present study had some limitations. First, the number of study subjects was small. Despite the statistically significant differences in the SBTT, image quality and amount of water in the small bowel were observed among the three study groups. Second, our results may be biased, because females were excluded from this study. Some studies have reported an influence of gender on the gastrointestinal motility, with the transit time in females tending to be longer than that in males [26–30].

## 5. Conclusion

Our study shows that lubiprostone significantly decreases the SBTT and improves the visualization of the small bowel during CE. We also confirm that lubiprostone induces water secretion into the small bowel lumen by capsule endoscopy.

## Figures and Tables

**Table 1:** Characteristics of the subjects.

Subjects number	6
Age	35.8 (29–50)
Sex (male/female)	6/0
Height (cm)	172.5 (165–178)
Weight (kg)	61.8 (52–70)
Body-mass index (kg/m^2^)	20.8 (16.7–22.2)
Drinking history	5
Smoking history	2

Median (minimum–maximum).

**Table 2:** Transit time of the capsule endoscope.

	P-P regimen	L-P regimen	P-L regimen	*P* value
Gastric transit time (min)	22.5 (9–160)	40 (4–122)	57.5 (15–78)	0.846
Small bowel transit time (min)	178.5 (117–407)	122.5 (27–282)	110.5 (11–331)	0.042

Median (minimum–maximum).

*P* values were calculated by the Friedman test.

**Table 3:** Scores for image quality score and amount of water, average ± standard deviation (SD).

	P-P regimen	L-P regimen	P-L regimen	*P* value
Median image quality score	2.88 ± 1.35	3.56 ± 0.56	3.76 ± 0.85	<0.001
Median amounts of water	1.66 ± 1.65	3.13 ± 1.64	2.60 ± 1.29	<0.001

Average ± standard division.

*P* values were calculated by the Friedman test.

## Abbreviations

GTT: Gastric transit time
SBTT: Small bowel transit time
P-P regimen: Placebo-placebo regimen
P-L regimen: Placebo-lubiprostone regimen
L-P regimen: Lubiprostone-placebo regimen
CE: Capsule endoscopy
PEG: Polyethylene glycol.

## References

[1] Lewis B. S., Swain P. (2002). Capsule endoscopy in the evaluation of patients with suspected small intestinal bleeding: results of a pilot study. *Gastrointestinal Endoscopy*.

[2] Eliakim R., Fischer D., Suissa A. (2003). Wireless capsule video endoscopy is a superior diagnostic tool in comparison to barium follow-through and computerized tomography in patients with suspected Crohn's disease. *European Journal of Gastroenterology and Hepatology*.

[3] Hartmann D., Schilling D., Bolz G. (2003). Capsule endoscopy versus push enteroscopy in patients with occult gastrointestinal bleeding. *Zeitschrift fur Gastroenterologie*.

[4] Costamagna G., Shah S. K., Riccioni M. E. (2002). A prospective trial comparing small bowel radiographs and video capsule endoscopy for suspected small bowel disease. *Gastroenterology*.

[5] Appleyard M., Glukhovsky A., Swain P. (2001). Wireless-capsule diagnostic endoscopy for recurrent small-bowel bleeding. *The New England Journal of Medicine*.

[6] Ou G., Svarta S., Chan C., Galorport C., Qian H., Enns R. (2014). The effect of chewing gum on small-bowel transit time in capsule endoscopy: a prospective, randomized trial. *Gastrointestinal Endoscopy*.

[7] Niv Y., Niv G. (2004). Capsule endoscopy: role of bowel preparation in successful visualization. *Scandinavian Journal of Gastroenterology*.

[8] Dai N., Gubler C., Hengstler P., Meyenberger C., Bauerfeind P. (2005). Improved capsule endoscopy after bowel preparation. *Gastrointestinal Endoscopy*.

[9] Ben-Soussan E., Savoye G., Antonietti M., Ramirez S., Ducrotté P., Lerebours E. (2005). Is a 2-liter PEG preparation useful before capsule endoscopy?. *Journal of Clinical Gastroenterology*.

[10] van Tuyl S. A. C., den Ouden H., Stolk M. F. J., Kuipers E. J. (2007). Optimal preparation for video capsule endoscopy: a prospective, randomized, single-blind study. *Endoscopy*.

[11] Viazis N., Sgouros S., Papaxoinis K. (2004). Bowel preparation increases the diagnostic yield of capsule endoscopy: a prospective, randomized, controlled study. *Gastrointestinal Endoscopy*.

[12] Leung W. K., Chan F. K. L., Fung S. S. L., Wong M.-Y., Sung J. J. Y. (2005). Effect of oral erythromycin on gastric and small bowel transit time of capsule endoscopy. *World Journal of Gastroenterology*.

[13] Kalantzis C., Triantafyllou K., Papadopoulos A. A. (2007). Effect of three bowel preparations on video-capsule endoscopy gastric and small-bowel transit time and completeness of the examination. *Scandinavian Journal of Gastroenterology*.

[14] Cuppoletti J., Malinowska D. H., Tewari K. P. (2004). SPI-0211 activates T84 cell chloride transport and recombinant human ClC-2 chloride currents. *The American Journal of Physiology—Cell Physiology*.

[15] Postgate A., Tekkis P., Patterson N., Fitzpatrick A., Bassett P., Fraser C. (2009). Are bowel purgatives and prokinetics useful for small-bowel capsule endoscopy? A prospective randomized controlled study. *Gastrointestinal Endoscopy*.

[16] Camilleri M., Bharucha A. E., Ueno R. (2006). Effect of a selective chloride channel activator, lubiprostone, on gastrointestinal transit, gastric sensory, and motor functions in healthy volunteers. *American Journal of Physiology: Gastrointestinal and Liver Physiology*.

[17] Kanda Y. (2013). Investigation of the freely available easy-to-use software “EZR” for medical statistics. *Bone Marrow Transplantation*.

[18] Johanson J. F., Ueno R. (2007). Lubiprostone, a locally acting chloride channel activator, in adult patients with chronic constipation: a double-blind, placebo-controlled, dose-ranging study to evaluate efficacy and safety. *Alimentary Pharmacology & Therapeutics*.

[19] Hussar D. A. (2006). New drugs: lubiprostone, ranolazine, and anidulafungin. *Journal of the American Pharmacists Association*.

[20] Fireman Z., Kopelman Y., Fish L., Sternberg A., Scapa E., Mahajna E. (2004). Effect of oral purgatives on gastric and small bowel transit time in capsule endoscopy. *Israel Medical Association Journal*.

[21] Endo H., Kondo Y., Inamori M. (2008). Ingesting 500 ml of polyethylene glycol solution during capsule endoscopy improves the image quality and completion rate to the cecum. *Digestive Diseases and Sciences*.

[22] Selby W. (2005). Complete small-bowel transit in patients undergoing capsule endoscopy: determining factors and improvement with metoclopramide. *Gastrointestinal Endoscopy*.

[23] Wei W., Ge Z. Z., Lu H., Gao Y. J., Hu Y. B., Xiao S. D. (2007). Effect of mosapride on gastrointestinal transit time and diagnostic yield of capsule endoscopy. *Journal of Gastroenterology and Hepatology*.

[24] Rabine J. C., Barnett J. L. (2001). Management of the patient with gastroparesis. *Journal of Clinical Gastroenterology*.

[25] Ramsbottom N., Hunt J. N. (1970). Studies of the effect of metoclopramide and apomorphine on gastric emptying and secretion in man. *Gut*.

[26] Ristvedt S. L., McFarland E. G., Weinstock L. B., Thyssen E. P. (2003). Patient preferences for CT colonography, conventional colonoscopy, and bowel preparation. *The American Journal of Gastroenterology*.

[27] Knight L. C., Parkman H. P., Brown K. L. (1997). Delayed gastric emptying and decreased antral contractility in normal premenopausal women compared with men. *The American Journal of Gastroenterology*.

[28] Parkman H. P., Harris A. D., Miller M. A., Fisher R. S. (1996). Influence of age, gender, and menstrual cycle on the normal electrogastrogram. *The American Journal of Gastroenterology*.

[29] Wedmann B., Schmidt G., Wegener M., Coenen C., Ricken D., Althoff J. (1991). Effects of age and gender on fat-induced gallbladder contraction and gastric emptying of a caloric liquid meal: a sonographic study. *American Journal of Gastroenterology*.

[30] Hutson W. R., Roehrkasse R. L., Wald A. (1989). Influence of gender and menopause on gastric emptying and motility. *Gastroenterology*.

